# Supplementary material for: The Intersection of Sexual Orientation, Substance Use, and Mental Health: Findings from Hints 5
Source: Healthcare (Basel). 2024 Oct 18;12(20):2083. doi: 10.3390/healthcare12202083 (PMC11507157; doi:10.3390/healthcare12202083)
Supplement: Supplementary file 1 [file healthcare-12-02083-s001.zip › healthcare-3194869-supplementary.pdf]

**Supplemental Material: Demographic Variables and Their Relationship with Sexual Orientation**

## S1. Introduction

Sexual orientation is a critical independent variable in studies examining lifestyle behaviours and mental health outcomes. To better understand how controlling for other demographic variables may influence results, this supplemental material provides an in-depth analysis of the relationships between sexual orientation and the following demographic variables: age, race, marital status, education, income sufficiency, and geographic area. By detailing these relationships, this material aims to clarify the context in which sexual orientation operates and how it interacts with other variables in the study.

## S2. Variables Overview

The variables examined in this analysis are as follows:

- **Sexual Orientation**: Heterosexual and Others (including gay, lesbian, bisexual, and other non-heterosexual orientations);
- **Age Group**: 18-34, 35-39, 40-44, and 45+;
- **Race**: Non-Hispanic White, Non-Hispanic Black or African American, Hispanic, Non-Hispanic Asian, and Non-Hispanic Other;
- **Marital Status**: Married, Divorced, and Single;
- **Education**: Less than High School, High School Graduate, Some College, and College Graduate or More;
- **Income Sufficiency**: Living comfortably, Getting by, Finding it difficult, and Finding it very difficult;
- **Area**: Metropolitan, Micropolitan, Small Town, and Rural.

## S3. Analysis of Relationships Between Sexual Orientation and Demographic Variables

### S3.1. Age Group

LGBTQ individuals are disproportionately represented in the 18-34 age group compared to heterosexual individuals. This younger age profile among LGBTQ individuals may partially explain their higher rates of behaviours such as smoking and e-cigarette use, influenced by factors such as e-cigarette marketing and social

pressures. Controlling for age in the primary analysis could help reduce the apparent impact of sexual orientation on these behaviours.

**S3.2. Race**

LGBTQ individuals are more represented among non-Hispanic Black, Hispanic, and other racial minorities compared to heterosexual individuals, who are predominantly non-Hispanic White. These racial differences could influence study outcomes, as racial minorities often face additional social and economic stressors that may intersect with those related to sexual orientation. Therefore, controlling for race might clarify whether observed disparities are attributable to sexual orientation or to intersecting racial challenges.

**S3.3. Marital Status**

Research has shown that marital status is significantly associated with sexual orientation, with LGBTQ individuals being more likely to be single or divorced compared to their heterosexual counterparts. This marital status distribution might affect the mental health and lifestyle behaviours of LGBTQ individuals, as single or divorced status is often associated with less social support. Controlling marital status could help isolate the effects of sexual orientation from those related to marital circumstances.

**S3.4. Education**

Gay men and lesbian women are more likely to have higher levels of education compared to bisexual individuals. This educational disparity may impact income sufficiency and stress, potentially influencing health behaviours. Including education as a control variable helps clarify whether differences in health behaviours are attributable to sexual orientation or educational attainment.

**S3.5. Income Sufficiency**

Income sufficiency is closely tied to sexual orientation, with LGBTQ individuals reporting more financial difficulty. Financial strain is a known contributor to stress and unhealthy behaviours, such as smoking and e-cigarette use. Controlling for income sufficiency in the primary analysis could help determine if observed disparities are due to economic challenges rather than sexual orientation per se.

**S3.6. Area**

Urban settings may offer more resources for LGBTQ individuals but may also come with higher social stressors, such as discrimination. Controlling for location helps to disentangle the effects of urban versus rural living from those directly related to sexual orientation.

**S4. Conclusion**

This supplemental analysis highlights the significant associations between sexual orientation and various demographic variables. Understanding these relationships is crucial for interpreting how controlling for these variables might influence study outcomes. The findings suggest that demographic factors such as age, race, marital status, education, income, and geographic area play a substantial role in shaping the experiences of LGBTQ individuals, particularly regarding health behaviours such as smoking and e-cigarette use. Controlling these variables in the main analysis is essential for accurately assessing the impact of e-cigarette use on health outcomes.
